# Supplementary figures and images for: DWI Metrics Differentiating Benign Intraductal Papillary Mucinous Neoplasms from Invasive Pancreatic Cancer: A Study in GEM Models
Source: Cancers (Basel). 2022 Aug 20;14(16):4017. doi: 10.3390/cancers14164017 (PMC9406679; doi:10.3390/cancers14164017)

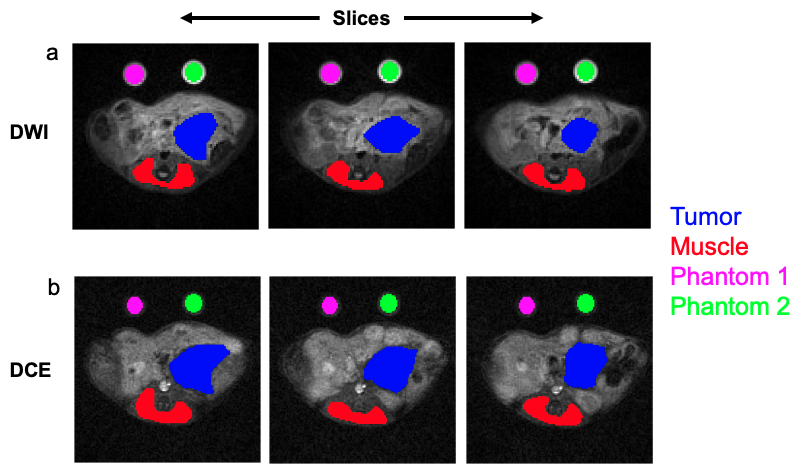

Supplement: Supplementary file 1 [file cancers-14-04017-s001.zip › supp_fig_S1.png]

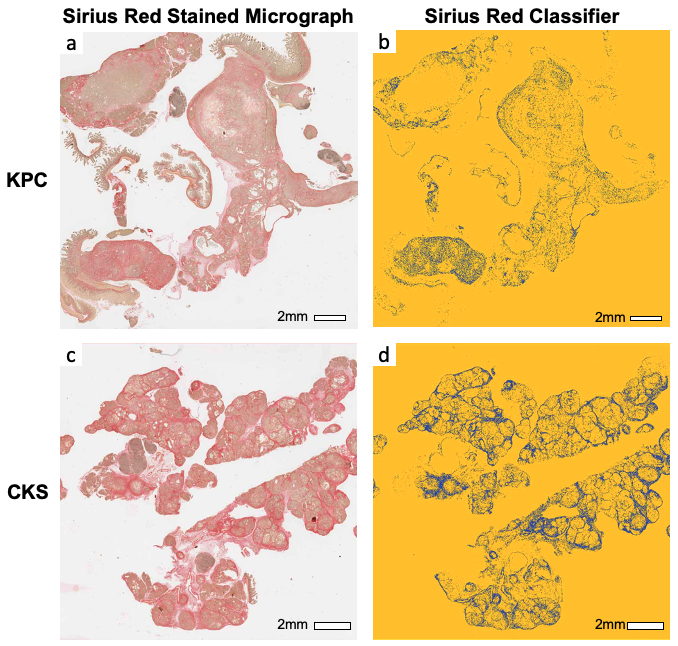

Supplement: Supplementary file 1 [file cancers-14-04017-s001.zip › supp_fig_S2.png]

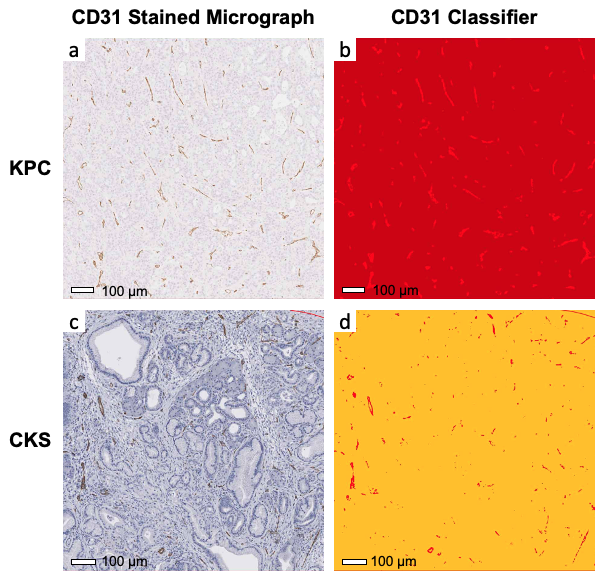

Supplement: Supplementary file 1 [file cancers-14-04017-s001.zip › supp_fig_S3.png]

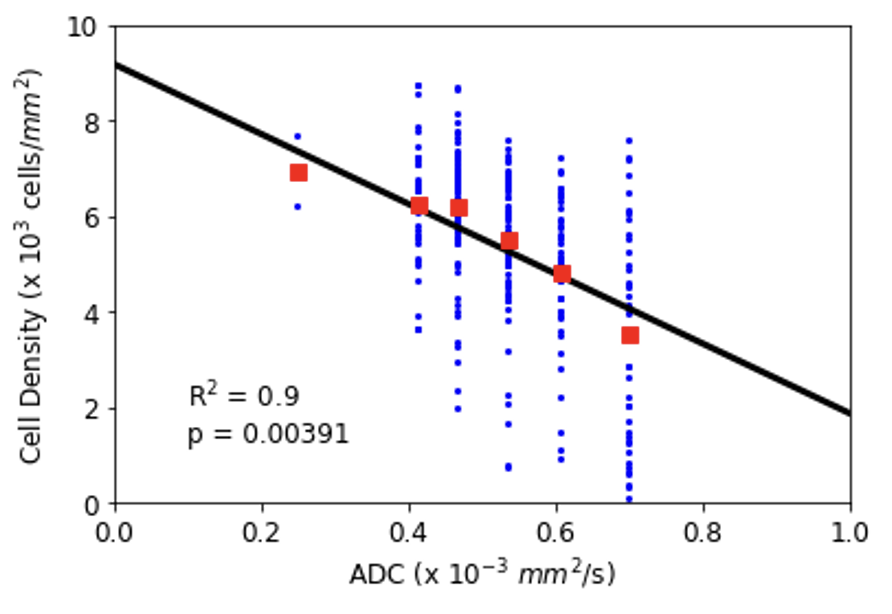

Supplement: Supplementary file 1 [file cancers-14-04017-s001.zip › supp_fig_S4.png]
